# The development of the biological soil crust regulates the fungal distribution and the stability of fungal networks

**DOI:** 10.3389/fmicb.2024.1347704

**Published:** 2024-05-30

**Authors:** Qian Liu, Shuping Zhou, Bingchang Zhang, Kang Zhao, Fei Wang, Kaikai Li, Yali Zhang

**Affiliations:** ^1^College of Life Sciences, Shanxi Normal University, Taiyuan, China; ^2^Geographical Science College, Shanxi Normal University, Taiyuan, China

**Keywords:** biological soil crusts, fungal community, fungal biodiversity, network analysis, desert soil

## Abstract

The heterogeneous composition of fungi plays an indispensable role in the foundation of the multifunctionalities of ecosystems within drylands. The precise mechanisms that govern fluctuations in soil fungal assemblages in dryland ecosystems remain incompletely elucidated. In this study, biological soil crusts (biocrusts) at different successional stages in the Gurbantunggut Desert were used as substrates to examine the characteristics and driving factors that influence fungal abundance and community dynamics during biocrust development using qPCR and high-throughput sequencing of the ITS2 region. The findings showed that the physicochemical properties changed significantly with the development of biocrusts. In particular, total nitrogen increased 4.8 times, along with notable increases in ammonium, total phosphorus (2.1 times) and soil organic carbon (6.5 times). Initially, there was a rise in fungal abundance, which was subsequently followed by a decline as the biocrust developed, with the highest abundance detected in lichen crust (2.66 × 10^7^ copies/g soil) and the lowest in bare sand (7.98 × 10^6^ copies/g soil). Ascomycetes and Basidiomycetes emerged as dominant phyla, collectively forming 85% of the fungal community. As the biocrust developed, noticeable alterations occurred in fungal community compositions, resulting from changes in the relative proportions of Dothideomycetes, Lecanoromycetes and unclassified ascomycetes. Nitrogen, phosphorus, organic carbon content, and pH of biocrusts were identified as direct or indirect regulators of fungal abundance and community structure. The complexity of fungal networks increased as biocrusts developed as revealed by network analysis, but reduced in the stability of fungal communities within algal and lichen crusts. Keystone species within the fungal community also underwent changes as biocrust developed. These results suggested that shifts in interspecies relationships among fungi could further contribute to the variation in fungal communities during the development of biocrusts.

## 1 Introduction

The maintenance of ecosystem multifunction, such as degradation of organic materials, elemental cycle and primary plant production, relies heavily on the pivotal contributions of microbial communities (Van Der Heijden et al., 2008; Bardgett and van der Putten, 2014). The formation and influencing mechanisms of microbial diversity are of great concern around the world. Desert soils also have considerable microbial diversity, due to the strong environmental adaptability of microorganisms (Leung et al., 2020; Zhou et al., 2020). These microbial communities are the main drivers of the biogeochemical cycles, such as carbon, nitrogen, and phosphorus, as well as the water and soil conservation in dryland ecosystem (Eldridge et al., 2020; Li et al., 2021).

Biological soil crusts (hereafter biocrusts) that cover approximately 12% of the global terrestrial surface and 28.7% of the Gurbantungut desert in China (Zhang et al., 2007; Rodriguez-Caballero et al., 2018) served as an exceptional resource for conducting thorough investigations into the variability of microbial community composition. Biocrusts play a crucial role in the cycling of carbon and nitrogen in desert ecosystems and represent significant reservoirs of soil organic carbon and nitrogen (Yan-Gui et al., 2013; Zhao et al., 2016). They also play vital roles in the global phosphorus cycle (Qi et al., 2021). The biocrusts are formed by cyanobacteria, eukaryotic algae, bacteria, fungi, lichen, moss and soil particles and develop primarily through different successional stages such as bare soil and algal, lichen, and moss crust (Belnap, 2003). The typical characteristics are the regular succession of physicochemical properties and microbial community with the development of biocrusts (Liu et al., 2017, 2020; Li et al., 2021). It is widely accepted that changes in the physicochemical properties of biocrusts affect the diversity and composition of microbial communities (Philippot et al., 2023). Environmental factors account for 10.3–22.8% of the variation in bacterial communities (Su et al., 2020) and 37.1% in cyanobacterial and eukaryotic algal community variations (Zhao et al., 2021).

Within dryland soils, fungi constitute a crucial element of microbial communities, and the diversity of these community correlates positively with the multifunctionality of ecosystems (Hu et al., 2021). Beyond their role in the organic matter degradation, fungi contribute significantly to nitrogen transformation and carbon sequestration in dryland soils (Carvajal Janke and Coe, 2021; Wang et al., 2022). In biocrusts, fungal diversity also affects the execution of their ecological functions (States et al., 2003). Fungi not only bind to particles through their filamentous form and extracellular polymers, but also improve soil fertility through symbiosis with algae (Zhou et al., 2020). Soil fungal communities in dryland regions exhibit responses to a variety of environmental factors. Studies show that decreased precipitation, nitrogen deposition, dry-wet cycles, and drought negatively impacted fungal diversity, while increased CO_2_ concentration, warming, and increased precipitation have positive effects (Hu et al., 2021). Physicochemical factors, mainly the soil organic carbon, explained up to 60% of the variation in fungal community composition of the biocrusts (Zhang et al., 2018, 2022). However, controlled experiments *in situ* have shown that nitrogen deposition and enhanced precipitation exerts minimal influence on the fungal community structure (Gao et al., 2021; Liu et al., 2021), adding to the uncertainty of key environmental factors in the variation of the fungal community.

Interspecific relationships, which are included in determinism together with environmental filtering (Stegen et al., 2013; Zhou and Ning, 2017), are also an influential element shaping the assembly of the microbial community. Species in ecosystems was not independent entities but rather come together to create intricate networks through direct or indirect interactions with each other. Due to the high diversity of microorganisms in the environment, studying complex interactions among species using paired culture experiments is challenging (Layeghifard et al., 2017). Hence, there is a growing utilization of network analysis to scrutinize the potential species interrelationships within ecosystems or the potential coexistence patterns among species (Feng et al., 2022). Despite its inability to precisely delineated microorganism relationships, co-occurrence network analysis offered evidence of potential coexistence patterns, extending from paired taxonomic groups to inter-domain communities (Faust, 2021; Neu et al., 2021). By comparing the topological parameters of microbial networks under different treatments, it was possible to uncover the disparities in complexity and stability among microbial communities (Zhou et al., 2020; Yuan et al., 2021). This offers an effective method for an in-depth analysis of how the development of biocrusts influences the structure of fungal communities.

The aim of this study is to explore the distribution characteristics of fungal communities from the perspective of environmental factors and the interspecific relationships with the development of biocrusts. We hypothesize that variations in physicochemical properties and interactions between species are key factors influencing the distribution of fungal communities with the development of biocrusts. To investigate this, this study used a typical developmental sequence in the Gurbantunggut Desert, including bare sand, algal crust, lichen crust and moss crust as research subjects. The distributional pattern of the fungal community was analyzed using quantitative fluorescence PCR and high-throughput sequencing of the internal transcribed spacer (ITS) of the fungi. Furthermore, network analysis was used to interpret the response of fungal communities to the development of biocrusts.

## 2 Materials and methods

### 2.1 Site description and soil sample collection

As our previous study described (Zhao et al., 2024), the research area is located in the western part of the Gurbantunggut Desert (44°15′15.75″ N, 87° 40′27.55″ E, Supplementary Figure S1), near the Yizhan petroleum extraction station. The surface of the dunes in this area is mainly covered with well-developed biocrusts, with lichen and moss crusts present in the interdune lowlands. Due to human activities such as oil extraction and grazing, these biocrusts have been disturbed (Zhang et al., 2007), and areas of bare sand and newly developed algal crusts are also found in the interdune lowlands. Therefore, all the four types of biocrusts existed in the same interdune area. Insights into the biocrust landscape were provided by an earlier study conducted in the Gurbantunggut Desert (Xiaobing Zhou and Yuanming, 2021).

In June 2021, nine interdune lowlands spaced at least 1.0 km apart were randomly selected within the study area. The placement of one of the interdune lowlands was depicted in Supplementary Figure S1. Each of these lowlands was surveyed, and samples of bare sand, algal crust, lichen crust, and moss crust were gathered through visual identification. The collection depth for each sample ranged from 0 to 2 cm. To minimize spatial heterogeneity, five samples of each stage of biocrusts were collected in each lowland and then mixed to form a replicate for this study. Thus, a total of nine replicates were obtained for each biocrust developmental stage. Every sample obtained was separated into two subsamples: one was preserved at −80°C for the extraction of soil DNA, while the other was employed to assess physicochemical properties. The soil moisture content measured by the drying method showed that the samples collected in this study were devoid of moisture.

### 2.2 Determination of soil physicochemical properties

Soil physicochemical factors were measured using standard methods (Tan, 2005). Soil organic carbon (SOC) was determined using the K_2_Cr_2_O_7_ oxidation method. Total nitrogen (TN) was measured using the Kjeldahl method and the ammonium nitrogen and nitrate nitrogen contents were determined using a 1 M KCl extraction followed by analysis with an automated discrete analyzer (AQ2 +, SEAL Analytical Inc., England). Total phosphorus (TP) was determined using the NaOH Melting-Mo Te Sc Colorimetry method, and available phosphorus (AP) was measured using the 0.5 mol/L NaHCO_3_ Leaching-Mo Te Sc Colorimetry. The pH of the samples was determined using a pH meter (Sartorius PB-10, Germany) with a soil-to-water ratio of 1:2.5.

### 2.3 DNA extraction and quantification of fungal ITS

Genomic DNA was extracted from 0.25 g of each soil sample using the SPINeasy soil DNA kit (MP Biomedicals, United States). The concentration and purity of the extracted DNA were assessed using a NanoDrop ND00c spectrophotometer (NanoDrop Technologies, Wilmington, DE, United States). To analyze the abundance characteristics of the fungi in biocrusts, quantitative PCR (qPCR) of the ITS2 region of the fungi was performed using the QuantStudio3 real-time PCR system (ThermoFisher Scientific, MA, United States). The primers used for this were gITS7 and ITS4 (Ihrmark et al., 2012). The volume of the PCR reaction was μl, containing 1 μL of DNA (~10 ng), 1 μL of 10 μM primers, and 10 μL SYBR Green Premix (Takara, Japan). The PCR cycling conditions were 95 ° C for 5 min, followed by 56 ° C for 30 s, and 72 ° C for 30 s. Fluorescence data were collected at the end of the annealing phase. A standard curve (ranging from 10 to 10^8^) was generated using plasmids containing the ITS to calculate the number of copies of the ITS in the samples.

### 2.4 Amplicon sequencing and bioinformatics analysis of ITS2 region

The sequencing of ITS2 region and the processing of sequences were carried out by the Beijing Biomarker Sequencing Platform, using the same primers as in quantitative PCR. The basic process of sequencing and analysis was as follows: Amplicons were sequenced on the Illumina Novaseq 6000 platform using a paired-end mode (2 × 250 base pairs). Low-quality sequences were filtered using Trimmomatic v0.33 (sliding window: 50:) (Bolger et al., 2014). Paired-end sequences were merged using Usearch v10 (UPARSE, 13). Chimeric sequences were removed using the UCHIME software (Edgar et al., 2011). For the high-quality ITS sequences obtained, the operational taxonomic units (OTU) were delineated at a 97% similarity threshold using Usearch v10 (UPARSE, 13). Representative sequences for each OTU were selected and genetically analyzed by aligning with fungal ITS sequences from the UNITE database (Nilsson et al., 2019). To compensate for differences in sequencing depth, the number of ITS sequences in each sample was flattened to 56,214 in this study.

### 2.5 Statistical analysis

This study focused on fungal communities in bare sand, algal, lichen and moss crusts, analyzing the influence of environmental properties and the interspecific relationship on the variation of the fungal community. Soil factors included soil pH, total nitrogen (TN), total phosphorus (TP), available phosphorus (AP), soil organic carbon (SOC), ammonium nitrogen, nitrate nitrogen, and C/N/P ratios (calculated using the content of SOC, TN, and TP). The R software (version 4.2.0) was utilized for data analysis. The variations of soil physicochemical properties in different successions of biocrusts were evaluated by one-way ANOVA. Fungal abundance was normalized to log10 for statistical analysis. The Shannon index was calculated at the OTU level and the differences in the average fungal abundance and the Shannon index at different stages of development were analyzed using pairwise *t* tests. The relationships between both the fungal abundance and community and biocrust properties were analyzed using ordinary least squares (OLS). At first, all soil factors are selected as independent variables, and the variable that contributed significantly was selected, with estimated significance (P and R^2^). Redundancy analysis (RDA) was used to decipher the variation pattern of the composition of the fungal community with the development of biocrusts and the influence of physicochemical factors. Nonparametric multivariate analysis of variance (PERMANOVA) was used to evaluate differences in the composition of the fungal community. Venn diagrams were used to analyze the generalist and specialist of fungi in different stages of biocrusts.

To evaluate the direct and indirect effects of physicochemical factors of biocrust on fungal abundance and community structure, partial least squares path modeling (PLSPM) was used using the PLSPM package in R. The original PLSPM model was established based on the consensus of biological crust studies that the physicochemical properties of biocrusts significantly regulated the fungal community (Weber et al., 2016). The association between the physicochemical properties of soil also referenced our previous study in an arid grassland (Zhao et al., 2018). Therefore, the original PLSPM model (Supplementary Figure S2) was established based on the hypothesis that significantly altered N, P, SOC and pH had substantial effects on the fungal community of biocrusts. In this model, soil factors were classified into four latent variables: pH, Soil Organic Carbon (SOC), Nitrogen (TN and ammonium), and Phosphorus (TP and AP). The structure of the fungal community was indicated by the first two axes of the principal coordinate analysis (PCoA, 36.5% fungal community captured).

In this study, the iNAP platform^1^ (Feng et al., 2022) was used to construct fungal networks of different stages of biocrusts based on the random matrix theory (RMT) method. These networks were created to further assess the effects of biocrust development on community variations of fungi. Before data processing, OTUs with relative abundance greater than 1% and present in at least 7 replicates for each stage of development were selected for subsequent network construction. In addition to the correlation threshold (0.80) and the *p*-value, the fungal networks were constructed using the default parameters of the platform. The topological roles of the nodes were evaluated based on their connectivity within the module (Zi) and connectivity between modules (Pi) (Deng et al., 2012). The nodes were classified into four categories: peripherals (Zi ≤ 2.5, Pi ≤ 0.62), module hubs (Zi > 2.5, Pi ≤ 0.62), connectors (Zi ≤ 2.5, Pi > 0.62), and network hubs (Zi > 2.5, Pi > 0.62). The network and module hubs, as well as connectors, were considered keystone species in microbial networks. The stability of fungal networks at different stages of biocrust development was characterized by robustness when 50% of the nodes were randomly removed. Ultimately, the network’s edge and node attribute files produced were visualized via Gephi version 0.9.7.

## 3 Results

### 3.1 Differences in physicochemical properties and fungal communities with the development of biocrusts

There were notable differences in the physicochemical properties with the biocrust developed (Supplementary Tables S1, S2). Briefly, the pH decreased from 8.14 to 7.84 as the biocrust developed, and a significant difference was detected only between the bare sand soil and moss crust. Total nitrogen (TN) increased 4.8 times (from 0.2 to 0.96 g/kg soil), total phosphorus (TP) by 2.1 times (from 0.11 to 0.24 g/kg soil) and total organic carbon (SOC) by 6.5 times (from 1.78 to 11.72 g/kg soil). The ammonium increased as the succession of biocrusts that ammonium concentrations of the lichen and moss crusts exhibited significantly higher than the bare sand and the algal crust. The availability of phosphorus (AP) fluctuated with the development of biocrusts and no notable disparities in nitrate levels were observed across various successional stages of biocrusts.

### 3.2 Relations of fungal abundance and community with physicochemical properties

**Figure 1:**
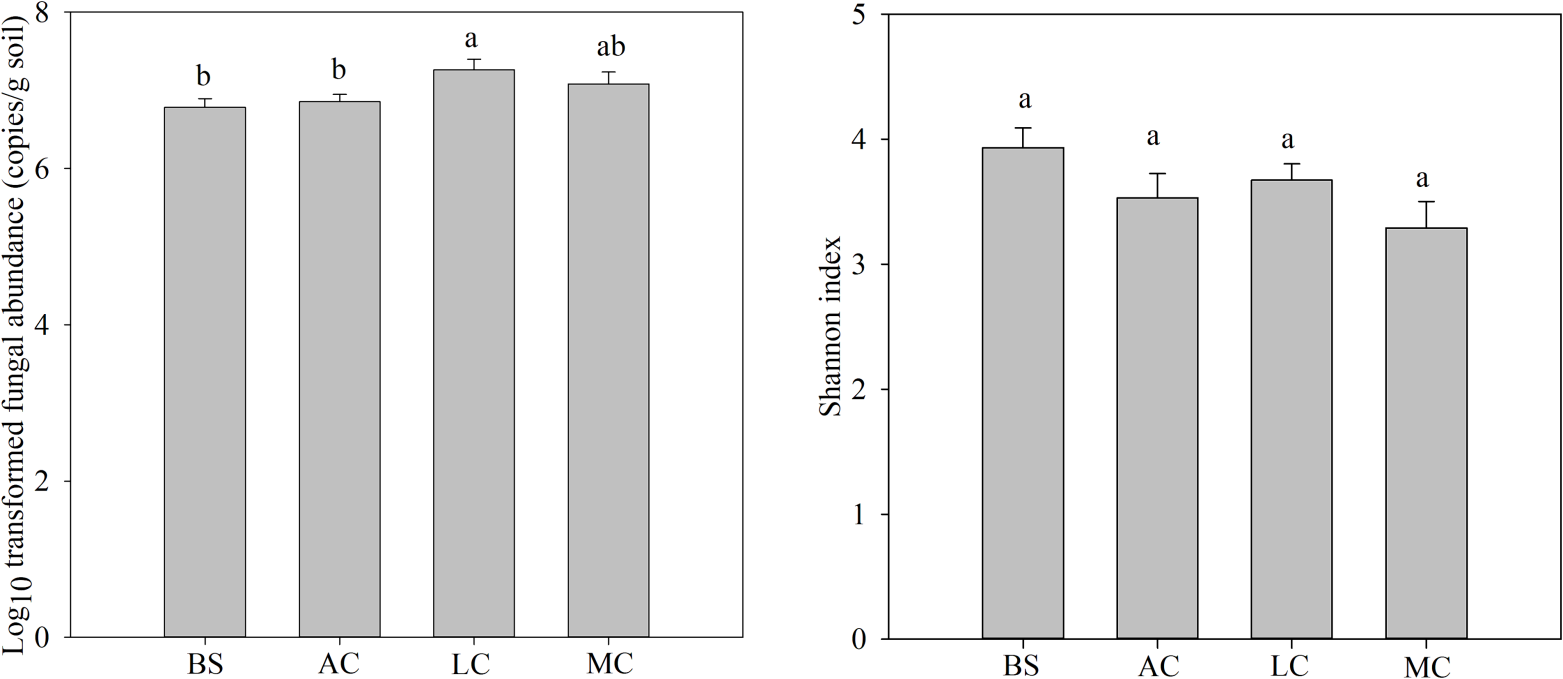
Differences in fungal abundance and community with the development of biocrusts. Different lowercase letters indicate significant differences between biocrust development stages (*p* < 0.05), and the values represent the average + standard error. BS, bare sand; AC, algal crust; LC, lichen crust; MC, moss crust.

The fungal abundance and community characteristics changed with the development of biocrusts (Figure 1; Supplementary Table S3). The highest fungal abundance was observed in the lichen crust, which was notably higher than that of bare sand and algal crust (*p* < 0.05). The fungal Shannon index showed a gradual decrease with the development of biocrusts. The moss crust exhibited the lowest fungal Shannon index among the samples analyzed.

**Figure 2:**
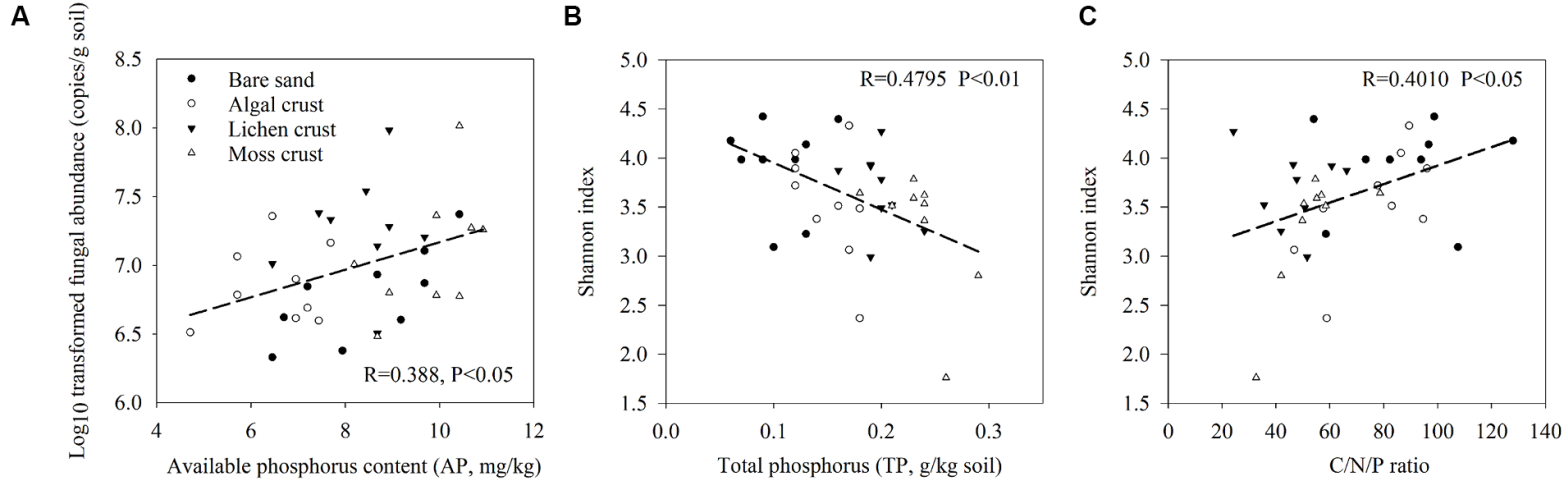
Regression analysis of fungal abundance, Shannon index and environmental factors. Pearson correlation was used to determine the significances. Significant regressions were shown. **(A)** Regression analysis of fungal abundance and available phosphorus (AP). **(B)** Regression analysis of the fungal Shannon index and total phosphorus (TP). **(C)** Regression analysis of the fungal Shannon index and C/N/P stoichiometric ratio. The stoichiometric ratio of C/N/P was calculated as SOC/TN/TP.

The distribution characteristics of the fungal communities with the development of biocrusts were related to variations in the physicochemical properties (Figure 2; Supplementary Table S4). The abundance of fungi increased with increasing phosphorus available in biocrusts. The fungal Shannon index was significantly associated with total phosphorus (TP) and stoichiometric ratios of C/N/P in biocrusts (*p* < 0.05). Specifically, the Shannon index of fungi showed a decrease with increasing TP and increased with increasing C/N/P (Figures 2B,C).

### 3.3 Variational characteristics in the fungal community and the effects of environmental factors

**Figure 3:**
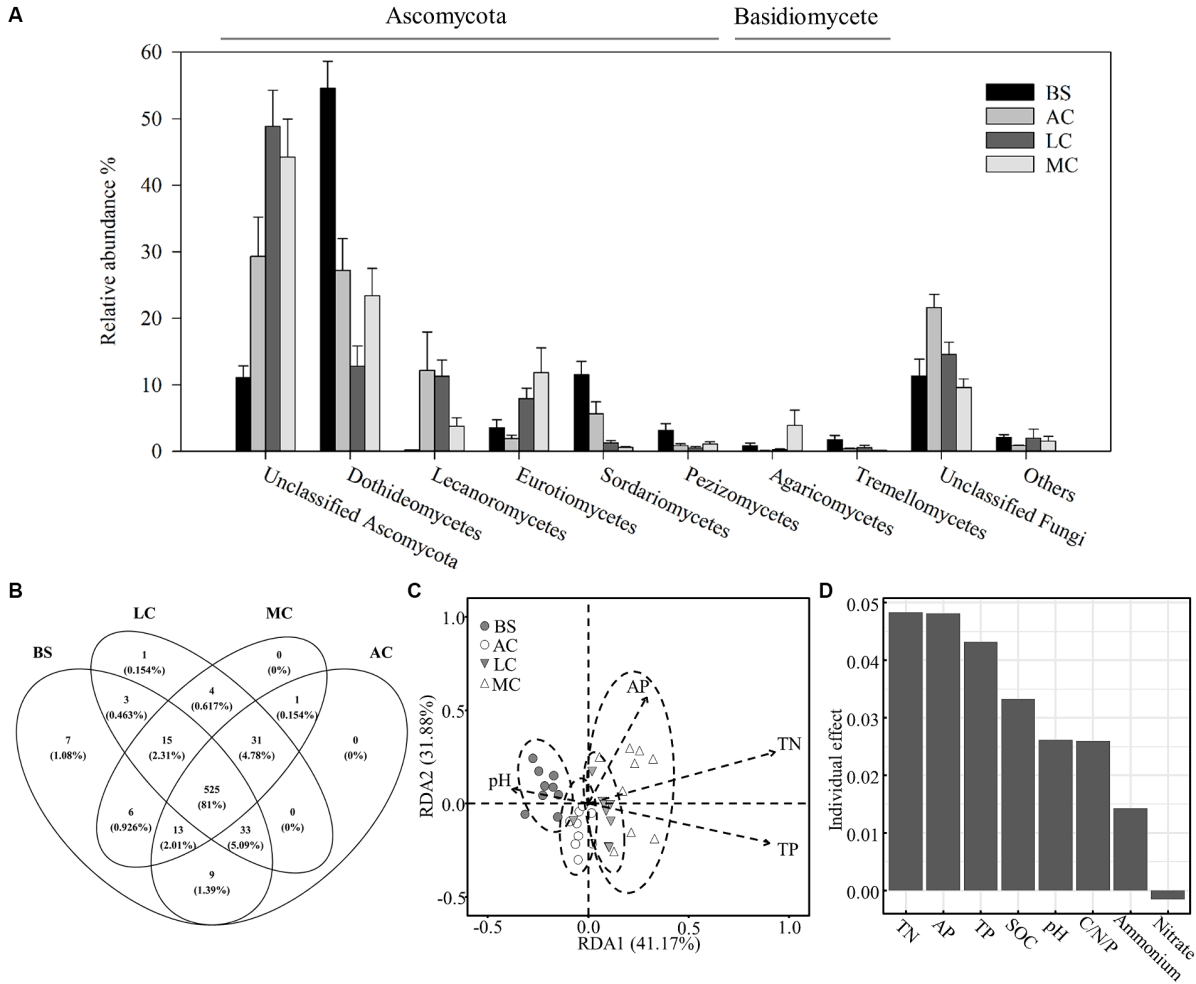
Differences in the composition and structure of the fungal community with the development of biocrusts and the influence of environmental factors. **(A)** Characteristics of the composition of the fungal community at the class level. **(B)** Distribution patterns of fungal OTUs at different stages of biocrusts. **(C)** The redundancy analysis (RDA) indicated differences between fungal communities at different stages of biocrusts and the main factors associated. Significant factors were employed in the RDA analysis through forward selection of environmental variables. **(D)** Independent effect of environmental factors on the variation of the fungal community. The package rdacca.hp. in R was used to calculate the independent effects of environmental factors on the variation of the fungal community. AP, available phosphorus; TN, total nitrogen; TP, total phosphorus; SOC, organic carbon, C/N/P was calculated by SOC/TN/TP. BS, bare sand; AC, algal crust; LC, lichen crust; MC: moss crust.

As shown in Figure 3, the main classes within the Ascomycota phylum (including Dothideomycetes, Lecanoromycetes and Eurotiomycetes) and Basidiomycota (including Agaricomycetes) dominated the fungal communities in the biocrusts, comprising approximately 85% of the fungal community. Approximately 14% of the fungal sequences remained unidentified. In the moss crust, Ascomycota exhibited the highest relative abundance among the observed fungal taxa (85.24%), and the lowest was observed in the algal crust (75.74%). Basidiomycota constituted 2.31–5.02% of the fungal communities, with the lowest in the lichen crust and the highest in the moss crust (Figure 3A). A total of 649 fungal OTUs were identified in the four stages of biocrusts. Of these, 525 OTUs were generalist in all stages of development, representing 81% of the fungal OTUs detected (Figure 3B). Nonparametric multivariate analysis of variance (PERMANOVA; Supplementary Tables S5, S6) and redundancy analysis (RDA, Figure 3C) further indicated that the structure of fungal communities changed significantly with the biocrust succession. Soil nitrogen (TN), phosphorus (TP and AP), and pH were key environmental factors that influenced the fungal community. These factors significantly affected the structure of fungal communities and also exerted comparatively greater individual impacts on the variation observed within the fungal community (Figure 3D).

**Figure 4:**
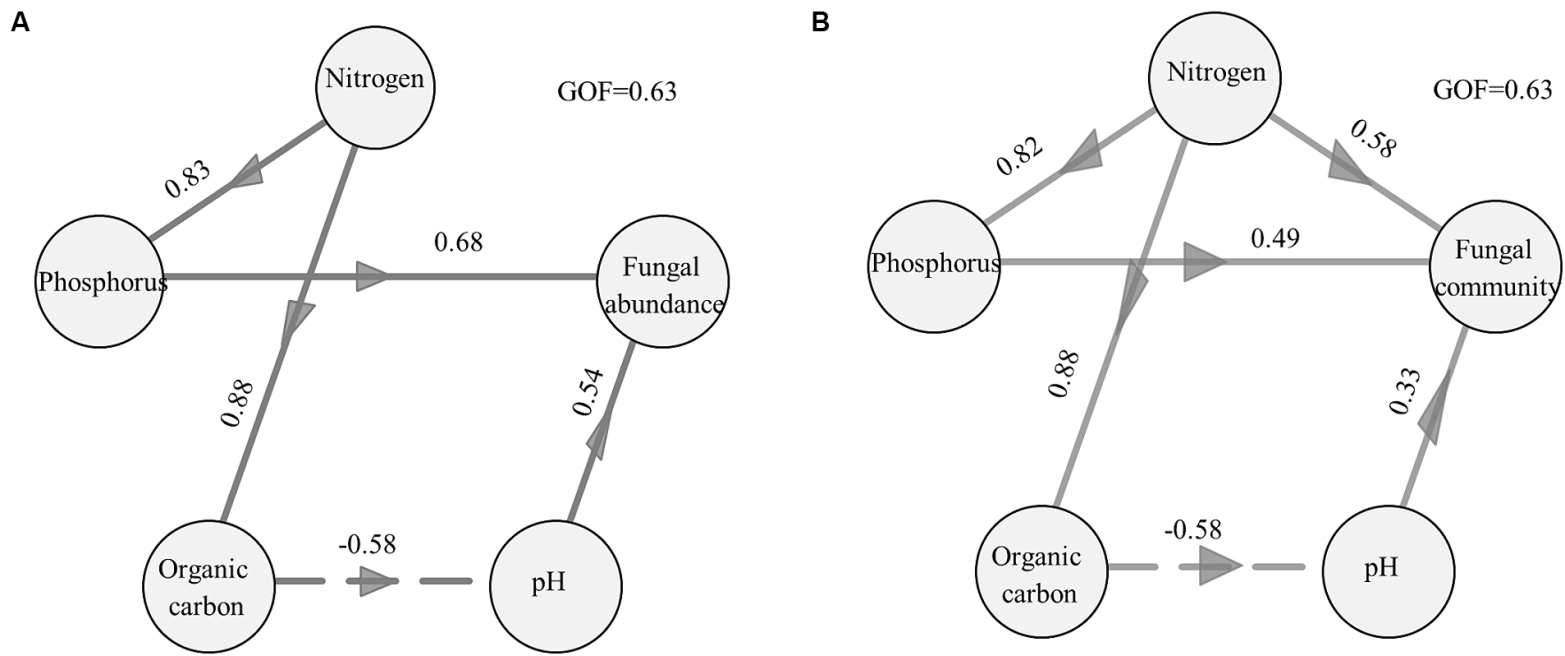
Direct and indirect effects of environmental factors on the abundance of fungi **(A)** and the structure of the fungal community **(B)** assessed by PLSPM. Nitrogen included total nitrogen (TN) and ammonium content, and phosphorus included total phosphorus (TP) and available phosphorus (AP). The first two axes of PCoA were used to indicate the fungal community. The coefficients were added near the path. Paths with significant effects were preserved. A pseudo-Goodness of Fit (GoF) was calculated measuring the reliability of the model.

The results of the PLSPM also revealed that changes in nitrogen, phosphorus, organic carbon, and pH in biocrusts directly or indirectly regulate the distribution of the fungal community (Figure 4; Supplementary Table S7). The phosphate content and pH directly affected the fungal abundance of biocrusts. The nitrogen and organic carbon content indirectly influenced fungal abundance within the biocrusts by modulating either the phosphorus content or pH values (Figure 4A). Nitrogen, phosphorus content, and pH were identified as key factors that directly controlled the fungal community structure of biocrusts. Furthermore, soil nitrogen content could indirectly influence the fungal communities by modulating phosphorus and organic carbon content (Figure 4B).

### 3.4 Network analysis of the fungal community

**Figure 5:**
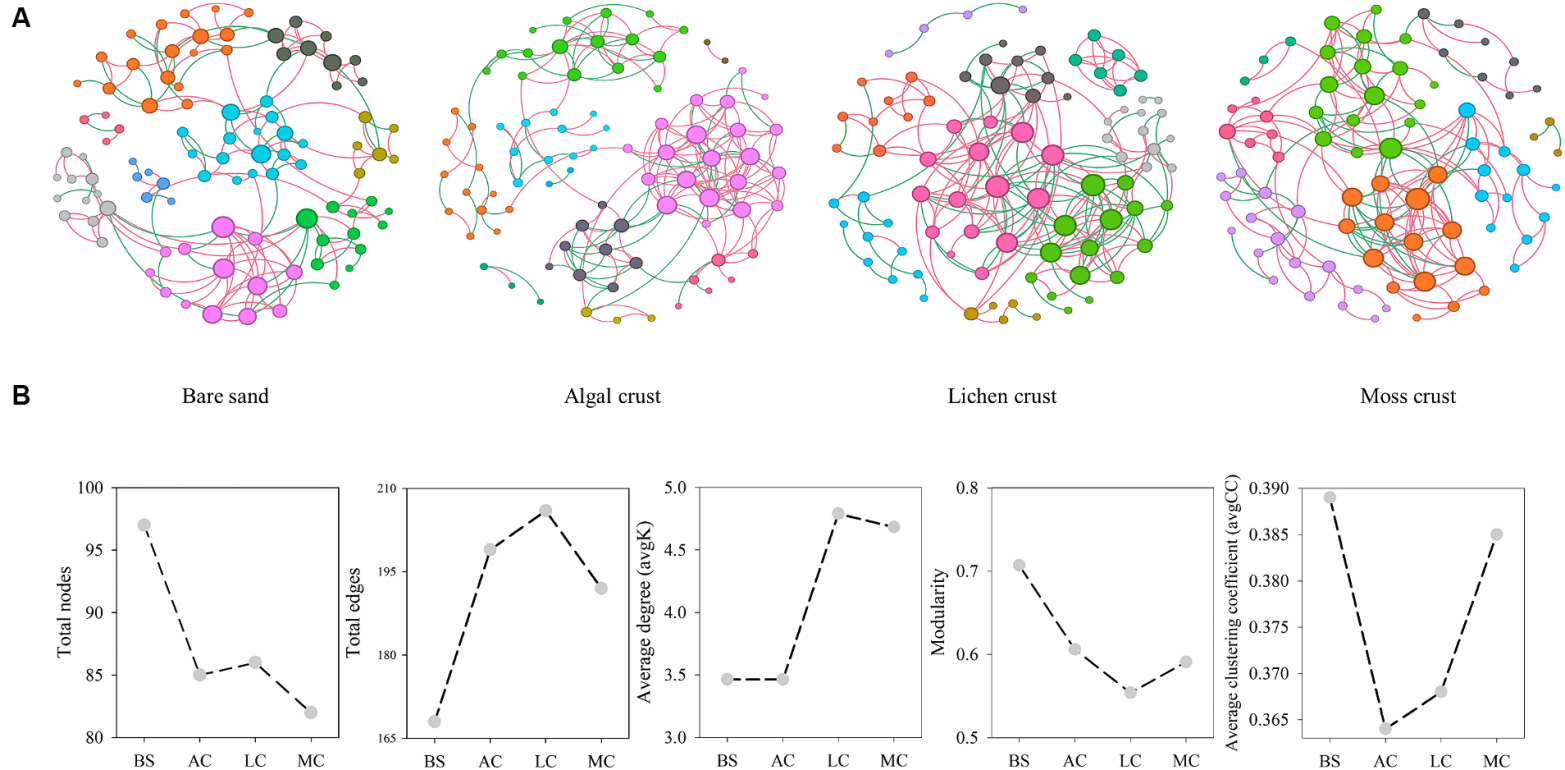
Patterns of fungal networks **(A)** and main topological properties **(B)** in different stages of biocrusts. The size of the node represented the degree. The node colors represented different modules. The edge color represented positive or negative correlations, with red representing positive correlations and green representing negative correlations. BS, bare sand; AC, algal crust; LC, lichen crust; MC, moss crust.

The occurrence of fungal networks depended on the development stages of the biocrusts (Figure 5; Table 1). The connectivity distribution curves of the fungal networks at different stages of biocrusts aligned well with the power law model (R^2^ of power law, Table 1). This alignment indicates that the networks exhibit scale-free properties. The modularity index, the average clustering coefficient and the average path length of the fungal networks in the four stages of development were higher than those of the random networks (Table 1). These results indicate that the networks exhibit characteristics of the small world and a modular structure.

**Table 1:** Topological parameters of the fungal network at different stages of biocrusts.

	Bare sand	Algal crust	Lichen crust	Moss crust
Total nodes	97	85	86	82
Total edges	168	199	206	192
*R*^2^ of power law	0.539	0.711	0.805	0.681
Negative edges percentage (%)	30.95	33.17	40.29	25.52
Average degree (avgK)	3.464	4.682	4.791	4.683
Average clustering coefficient (avgCC)	0.389	0.364	0.368	0.385
Average path length (GD)	5.200	5.908	3.935	4.598
Modularity	0.707	0.606	0.554	0.591
Density (D)	0.036	0.056	0.056	0.058
Connectedness (Con)	0.843	0.843	0.706	0.929
Robustness	0.287 ± 0.038b	0.275 ± 0.044bc	0.234 ± 0.041c	0.331 ± 0.037a
Random average clustering coefficient	0.001 ± 0.005	0.001 ± 0.004	0.004 ± 0.01	0.003 ± 0.008
Random average path length	3.712 ± 0.079	3.043 ± 0.077	3.009 ± 0.069	3.022 ± 0.074
Random modularity	0.508 ± 0.015	0.383 ± 0.012	0.38 ± 0.012	0.387 ± 0.013

Robustness was measured by the proportion of species remaining in the community after 50% of the nodes were randomly removed. The *t*-test was used to measure differences in the robustness of the networks. Different lowercase letters represent significant differences (*p* < 0.05).

The results of the networks’ topological properties further indicate that the development of biocrusts leads to increased complexity in fungal networks, with tighter connectivity between species (Table 1). This is mainly evidenced by a decrease in the total nodes but an increase in the total number of edges in the fungal networks as the development of biocrusts. Furthermore, with the development of biocrusts, the average degree and density of fungal networks increased, while the average path length decreased. These findings implied that the succession of biocrusts enhanced the interconnections among fungal species. As biocrusts develop, the relationships between fungal species can also potentially transform. In fungal networks, the percentage of negative correlations experienced a gradual rise from 30.95% (bare sand) to 40.29% (lichen crust). However, in moss crust, the proportion of negative correlations drops to 25.52%. The stability of fungal networks also changed regularly with the development of biocrusts. Robustness of the networks indicated that the stability of fungal communities first decreases and then increases as the biocrusts develop (Table 1; Supplementary Table S8). From the stage of bare sand to the stage of lichen crust, the robustness of the fungal networks decreased significantly. In contrast, as the moss crust developed, the robustness of the fungal networks increased significantly.

Differences in network structure can lead to alterations in the roles of species within the network. A total of seven key fungal species (keystone taxa) were identified, with 2 each in the bare sand, lichen and moss crusts, and 1 in the algal crust (Table 2). These keystone species include a module hub and six connectors. As biocrusts develop, key species within fungal communities also undergo succession. Specifically, species belonging to the Dothideomycetes class in the bare sand stage are replaced by species from the classes Eurotiomycetes, Lecanoromycetes and Agaricomycetes in the lichen and moss crust.

**Table 2:** Keystone taxa of fungal communities identified according to network analysis.

Developmental stages	Topological role	Phylum	Class	Order	Family	Genus
Bare sand	Connectors	Ascomycota	Dothideomycetes	Pleosporales	Phaeosphaeriaceae	
	Connectors	Ascomycota	Dothideomycetes	Pleosporales	Lentitheciaceae	*Keissleriella*
Algal crust	Module hubs	Ascomycota	Unclassified Ascomycota			
Lichen crust	Connectors	Ascomycota	Eurotiomycetes	Verrucariales	Verrucariaceae	*Placidium*
	Connectors	Ascomycota	Lecanoromycetes	Pertusariales	Megasporaceae	*Circinaria*
Moss crust	Connectors	Basidiomycota	Agaricomycetes	Corticiales	Unclassified Corticiales	*Marchandiomyces*
	Connectors	Ascomycota	Eurotiomycetes	Verrucariales	Verrucariaceae	*Verrucaria*

The keystone taxa are module hubs and connectors that are identified based on their within-module connectivity (Zi > 2.5) and among-module connectivity (Pi > 0.62).

## 4 Discussion

### 4.1 The impact of biocrust development on physicochemical properties

The development of biocrusts significantly increases nutrient content (Belnap et al., 2016; Xiao and Veste, 2017). This study also showed that the contents of total nitrogen (TN), ammonium, total phosphorus (TP), and soil organic carbon (SOC) increased significantly with the succession of biocrusts. There were significant differences in nutrient accumulation at different developmental stages of biocrusts, mainly due to the variable efficiency of nutrient absorption and enrichment in different types of biocrusts. Later stages of biocrust have stronger effects on nutrient accumulation and greater nutrient storage (Zhang et al., 2015). A substantial increase in total nitrogen and organic carbon during development is related to the carbon and nitrogen fixation capabilities of biocrusts (Li et al., 2021). A previous study indicated that the exclusive presence of BSC resulted in an elevation of potentially available P, attributed to heightened organic matter and organism activity within the ecosystem (Baumann et al., 2017). Our study also observed a significant increase in the phosphorus content with the development of biocrusts. Furthermore, this study found that soil pH gradually decreased as the biocrust developed, possibly due to acid substances secreted by microbes during the lichen and moss crusts (Leao et al., 2012; Kakeh et al., 2018).

### 4.2 Fungal communities respond to succession of biocrusts

The abundance of fungi increased as biocrust developed, reaching the highest level in the lichen crust. These findings aligned with the conclusions drawn from prior research conducted in the Gurbantunggut Desert (Zhang et al., 2018). Furthermore, fungal communities at different biocrust developmental stages showed a high level of similarity in species composition, showing that 81% of the species were generalists. This observation deviated somewhat from the widely held belief that microbial community similarity diminishes with increasing time and distance (Green et al., 2004; Martiny et al., 2006; Guo et al., 2018). The similarity in the fungal community composition between different types of biocrusts may be attributed to the limited sampling distance and the short-range dispersal of fungi within a small-scale geographical distance. Previous research suggests that eukaryotic community compositions show significant differences over geographic distances exceeding 1–3 km (Green et al., 2004; Martiny et al., 2006). In this study, all types of biocrusts existed in the same interdune lowlands, and the microgeographic scale was not sufficient to cause noteworthy variations which observed in the fungal community. Furthermore, short-distance migration of microbes with windblown sand in desert areas (Zhang et al., 2007) could increase the similarity in fungal composition. With the development of biocrusts, there are significant fluctuations in the relative abundance of the main fungal groups, leading to significant differences in the structure of the fungal communities. As the results showed, fungi from the Ascomycota and Basidiomycota phylum dominated in biocrusts at all stages of development. The result was consistent with previous studies in various desert areas such as the cold desert of Colorado and the Mu Us Sandland in China (Bates and Garcia-Pichel, 2009; Tian et al., 2021). Ascomycota maintained higher proportion at all stages of biocrusts, likely due to the melanin-rich cell walls of many species in this phylum, which protect against abiotic stresses such as dryness and high UV radiation in desert environments (Abed et al., 2013; Liu et al., 2017). The fluctuation in the relative abundance of Basidiomycota may be related to its predominantly saprotrophic or symbiotic characteristics (Lundell et al., 2014). With the development of biocrusts, the Shannon index of fungal communities gradually decreased. Low nutrient content, drought, and high UV stress in bare sand could induce most fungi into a dormant state, mitigating environmental stress and avoiding potential interspecific competition (Che et al., 2019; Coleine et al., 2022), resulting in higher α-diversity in bare sand. As biocrusts develop, changes in the microhabitat potentially intensified interspecific microbial competition, resulting in a decrease in α-diversity in later developmental stages of biocrusts. The increasing proportion of negative correlations in fungal networks with biocrust development, observed in this study, also reflects intensified competition between fungal species.

### 4.3 The effects of physicochemical properties on fungal communities

With the development of biocrusts, fungal communities undergo regular variations. It is widely believed that as biocrusts develop and soil nutrients accumulate, microbial activity and biomass increase correspondingly, leading to higher fungal abundance (Yu et al., 2012; Liu et al., 2020). Compared to early-stage biocrusts, well-developed biocrusts provide a more suitable microhabitat for fungal proliferation, such as higher carbon, nitrogen, and phosphorus content and water holding capacity (Xiao and Veste, 2017; Eldridge et al., 2020). Thus, well-developed biocrusts, such as lichen and moss crusts, offer better protection for fungi against environmental stress. This is a potential mechanism by which fungal abundance increases with the development of biocrusts in this study.

This study suggested that the distribution of the fungal community had associations with the physicochemical factors of the biocrusts. The development of biocrusts resulted in significant differences in soil carbon, nitrogen, phosphorus, and pH, altering the microenvironment of soil (Couradeau et al., 2016; Maier et al., 2018; Zhang et al., 2023). These environmental changes potentially led to differential responses in various fungal groups, which affected the structure of fungal communities in this study. Previous research in the Gurbantunggut Desert found that changes in the fungal community structure were found to be correlated with differences in the levels of organic carbon content, electrical conductivity and soil bulk density (Zhang et al., 2018). In desert ecosystems, the increased availability of organic substrates also stimulated fungal biomass and activity, leading to compositional differences in the community (Liu et al., 2013; Zhang et al., 2015). In this research, the content of organic matter was also an essential factor contributing to the fungal community variation. A study of soil arbuscular mycorrhizal fungi also found that their abundance and spore density increased with available phosphorus, but are inhibited by an excessively high soil phosphorus content (more than 50 mg/kg) (Ryan and Graham, 2002). Furthermore, the abundance and diversity of fungal soils are negatively correlated with soil pH (Davison et al., 2021). The phosphorus level (TP and AP) and pH played significant roles in driving variations within the fungal community with the development of biocrusts as this study showed. The observed variations in fungal communities throughout the development of biocrusts could be elucidated by the reaction of fungal communities to these pivotal physicochemical factors.

### 4.4 The development of biocrusts changed the fungal networks

Microbial networks can indicate beneficial or harmful coexistence relationships among microbes (Feng et al., 2019), which was an important factor that affected the assembly of the microbial community. The extent of interaction quantifies the influence of a species on the growth and survival of other organisms in the community, thus molding the community’s overall composition and stability (Hu et al., 2022). Assessing the differences in community stability and intricacy involved comparing the topological properties of microbial networks across various treatments (Zhou et al., 2020; Yuan et al., 2021). These characteristics provide an effective method to deeply analyze the mechanisms of fungal community formation and variation with the development of biocrusts. The results of this study showed that, compared to the bare sand stage, the fungal networks in the biocrusts become more interconnected. This is mainly reflected in the reduced number of nodes and the increased number of edges and the average degree in fungal networks, along with a decrease in the average path length. The lower nutrient content and stronger environmental stress in the bare sand stage could reduce interspecies interactions due to low fungal activity (Xu et al., 2022). As biocrusts developed, improvements in the microenvironment, such as increased nutrient content, provide more opportunities for fungal interspecies relationships, and the increase in nutrients could improve the complexity of the microbial networks (Suweis et al., 2015; Zhao et al., 2019). Furthermore, the predominance of positive correlations in different stages of biocrusts suggested that fungal communities may maintain their structure through cooperative interactions or coexist independently.

Greater complexity in a microbial network generally indicates a more robust community, which increases tolerance to extreme environmental changes (de Vries et al., 2018). However, as the results showed, there was a gradual decrease in the stability of fungal networks from bare sand to lichen crust, followed by a notable increase in the moss crust. The increase in negative correlations could be one of the main reasons for the decreased stability of fungal communities. As biocrusts developed to lichen crust, the proportion of negative correlations in fungal networks increases, reflecting an increase in harmful interspecies relationships (Feng et al., 2019). The decreased stability of fungal community could make them more sensitive to variations in physicochemical factors, thereby accelerating variations occurred in the fungal community. Furthermore, the succession of keystone taxa of fungal communities with the development of biocrusts could also influence the variation in community structure. Although the reliance on calculation-based methods and the absence of experimental evidence regarding interspecies interactions have decreased the accuracy of identifying keystone species through network analysis, this widely employed approach still offered valuable insights (Berry and Widder, 2014; Faust, 2021). In this study, keystone species in fungal networks changed with the development of biocrusts, which could be related to changes in soil physicochemical properties that influence growth strategies of different fungal groups (Xu et al., 2022). The prevailing perspective underscores the pivotal role of keystone taxa in microbial community structure maintenance, irrespective of their abundance (Banerjee et al., 2018; Herren and McMahon, 2018). Consequently, these altered keystone taxa may have influenced both the fungal community composition and the direction of variation observed in our study.

## 5 Conclusion

The fungal community changed notably with the development of biocrusts. The soil nutrient content (total phosphorus, total nitrogen, and organic carbon) and the pH value are the key factors that affect the distribution of fungi within biocrusts. The development of biocrusts increased the complexity of fungal networks. However, transformation in the interspecies relationships of fungi reduced the stability of fungal networks in algal and lichen crusts, potentially promoting variations in fungal communities within biocrusts.

## Data availability statement

The datasets presented in this study can be found in online repositories. The names of the repository/repositories and accession number(s) can be found below: NCBI - PRJNA1048333.

## Author contributions

QL: Investigation, Writing – original draft. SZ: Investigation, Writing – original draft. BZ: Conceptualization, Data curation, Funding acquisition, Investigation, Supervision, Writing – review & editing. KZ: Conceptualization, Data curation, Funding acquisition, Investigation, Supervision, Writing – review & editing. FW: Data curation, Writing – review & editing. KL: Investigation, Methodology, Writing – review & editing. YZ: Investigation, Methodology, Writing – review & editing.

## Footnotes

^1^ http://mem.rcees.ac.cn:8081
