# Supplementary material for: The development of the biological soil crust regulates the fungal distribution and the stability of fungal networks
Source: Front Microbiol. 2024 May 30;15:1347704. doi: 10.3389/fmicb.2024.1347704 (PMC11169694; doi:10.3389/fmicb.2024.1347704)
Supplement: Supplementary file 1 [file Data_Sheet_1.pdf]

Figure S1

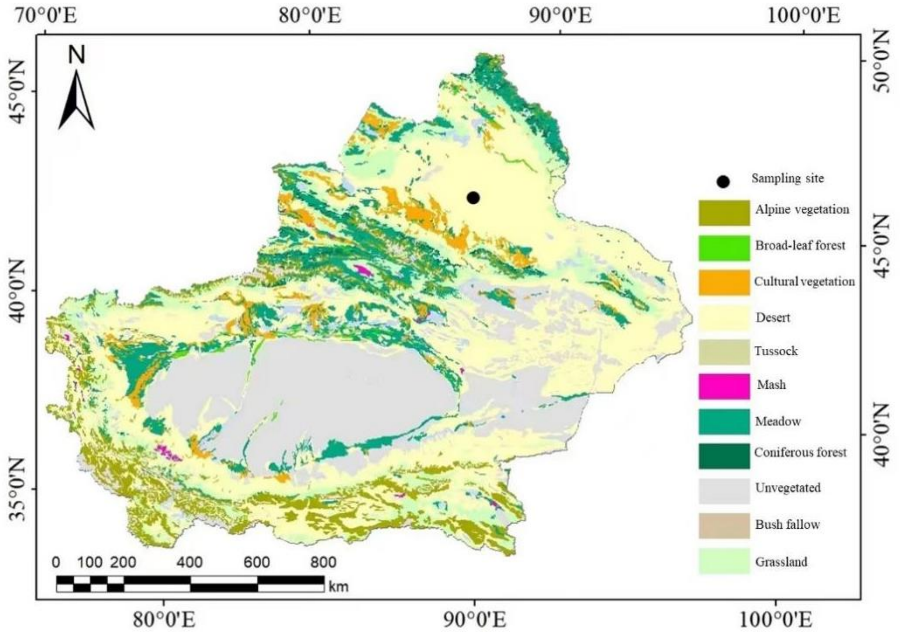

Figure S1 The sampling site diagram with the map of Xinjiang Uygur Autonomous Regio n, China. The vegetation dataset is provided by National Cryosphere Desert Data Center. (http://www.ncdc.ac.cn).

Figure S2

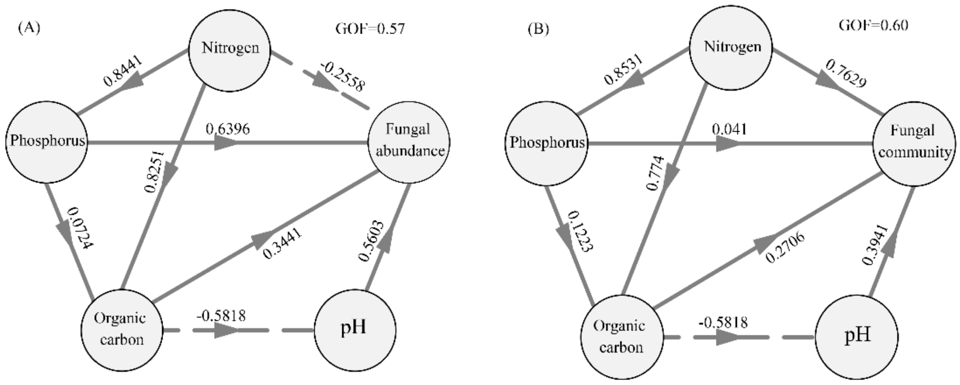

Figure S2 Original PLSPM models evaluating environmental factors on the abundance of fungi (A) and the structure of the fungal community (B). Nitrogen included total nitrogen (TN) and ammonium content, and phosphorus included total phosphorus (TP) and available phosphorus (AP). The first two axes of PCoA were used to indicate the fungal community. Paths with significant effects were preserved. A pseudo-Goodness of Fit (GoF) was calculated measuring the reliability of the model.

Table S1

Table S1 Physical and chemical properties at different developmental stages of biocrusts

Developmental stage	pH	TN (g/kg)	Nitrate (mg/kg)	Ammonium (mg/kg)	TP (g/kg)	AP (mg/kg)	SOC (g/kg)	C/N/P Stoichiometric ratio
Bare sand	8.14±0.06a	0.2±0.02d	1.58±0.24a	26.7±0.9b	0.11±0.01d	8.44±0.48b	1.78±0.2c	88.12±7.89a
Algal crust	8.19±0.08a	0.34±0.02c	1.45±0.1a	27.3±1.55b	0.15±0.01c	6.53±0.33c	3.8±0.33b	76.75±5.98a
Lichen crust	8.1±0.06a	0.57±0.02b	1.65±0.11a	32.73±2.07a	0.2±0.01b	8.32±0.32b	5.15±0.39b	47.23±4.2b
Moss crust	7.84±0.02b	0.96±0.06a	1.39±0.22a	34.45±2.16a	0.24±0.01a	9.79±0.32a	11.72±0.8a	53.16±4.19b

Values are average ± standard error, and different lowercase letters indicate significant differences between different stages of biocrust ($P < 0.05$). The C/N/P stoichiometric ratio was calculated as SOC/TN/TP.

Table S2 Comparison results of physicochemical properties at different stages of biocrust by One-way ANOVA

Comparison pair	pH	TN (g/kg)	Nitrate (mg/kg)	Ammonium (mg/kg)	TP (g/kg)	AP (mg/kg)	SOC (g/kg)	C/N/P Stoichiometric ratio
BS - AC	0.591	**0.009**	0.606	0.808	**0.001**	**0.001**	**0.006**	0.173
BS - LC	0.662	**0.000**	0.784	**0.020**	**0.000**	0.831	**0.000**	**0.000**
BS - MC	**0.002**	**0.000**	0.462	**0.004**	**0.000**	**0.014**	**0.000**	**0.000**
AC - LC	0.332	**0.000**	0.431	**0.035**	**0.001**	**0.002**	0.058	**0.001**
AC - MC	**0.000**	**0.000**	0.825	**0.007**	**0.000**	**0.000**	**0.000**	**0.007**
LC - MC	**0.006**	**0.000**	0.315	0.490	**0.007**	**0.008**	**0.000**	0.473

BS: bare sand, AC: algal crust, LC: lichen crust, MC: moss crust. The P values are shown in the table. Significant differences are marked in bold ($P < 0.05$).

Table S3 Differences in fungal abundance and Shannon index at different developmental stages assessed by Paired t-test

Comparison pair	Abundance			Shannon index		
	t	N_{total}	Two-tailed P-value	t	N_{total}	Two-tailed P-value
BS - AC	-0.634	18	0.544	1.806	18	0.109
BS - LC	-2.694	18	0.027*	1.167	18	0.277
BS- MC	-1.838	18	0.103	2.144	18	0.064
AC - LC	-2.623	18	0.030*	-0.736	18	0.483
AC - MC	-1.913	18	0.092	0.978	18	0.357
LC - MC	1.031	18	0.333	2.046	18	0.075

BS: bare sand, AC: algal crust, LC: lichen crust, MC: moss crust; *represents $P < 0.05$.

Table S4 Results of the regression analysis of fungal abundance, Shannon index and environmental factors.

Model summary	R	R^2	Adj R^2	$SE_{estimate}$		
Fungal abundance - Available phosphorus (AP)	0.3875	0.1502	0.1252	0.3811		
Fungal shannon index - Total phosphorus (TP)	0.4795	0.2299	0.2073	0.4966		
Fungal shannon index-C/N/P stoichiometric ratio	0.401	0.1608	0.1361	0.5184		
Coefficient		Coefficient	SE	t	P	
Fungal abundance - Available phosphorus (AP)	y0	6.1681	0.3437	17.9481	**<0.0001**	
Fungal shannon index - Total phosphorus (TP)	y0	4.427	0.2705	16.3688	**<0.0001**	
Fungal shannon index-C/N/P stoichiometric ratio	y0	2.9812	0.2598	11.4749	**<0.0001**	
Analysis of Variance		DF	SS	MS	F	P
Fungal abundance - Available phosphorus (AP)	Regression	2	1762.8826	881.4413	6069.7007	**<0.0001**
	Residual	34	4.9375	0.1452		
	Total	36	1767.8201	49.1061		
Fungal shannon index - Total phosphorus (TP)	Regression	2	470.7773	235.3887	954.5993	**<0.0001**
	Residual	34	8.3838	0.2466		
	Total	36	479.1612	13.31		
Fungal shannon index-C/N/P stoichiometric ratio	Regression	2	470.0244	235.0122	874.5374	**<0.0001**
	Residual	34	9.1367	0.2687		
	Total	36	479.1612	13.31		

Significant differences are marked in bold ($P < 0.05$).

Table S5 Differences in fungal communities assessed by PERMANOVA

	Algal crust	Lichen crust	Moss crust
Bare sand	0.20**	0.25**	0.22**
Algal crust		0.13**	0.23**
Lichen crust			0.16**

**represents $P < 0.01$，*represents $P < 0.05$.

Table S6 Results of the PERMANOVA analysis of the fungal community at different developmental stages of biocrusts

Comparison pair	Df_{total}	F.Model	R^2	p.value	p.adjusted	sig
BS - AC	18	4.01	0.20	0.001	0.002	**
BS - LC	18	5.31	0.25	0.001	0.002	**
BS - MC	18	4.48	0.22	0.001	0.002	**
AC - LC	18	2.31	0.13	0.004	0.004	**
AC - MC	18	4.69	0.23	0.001	0.002	**
LC - MC	18	3.09	0.16	0.003	0.004	**

BS: bare sand, AC: algal crust, LC: lichen crust, MC: moss crust. **represents $P < 0.01$.

Table S7 Analysis of significance of the path coefficient

Fungal abundance	Original	Mean.Boot	Std.Error	perc.025	perc.975
N->P	0.831793	0.834448	0.037895	0.753899	0.904961
N->SOC	0.8774	0.883496	0.024077	0.824338	0.9235
P->abundance	0.680534	0.677632	0.133998	0.429437	0.924935
SOC->pH	-0.58183	-0.57568	0.10229	-0.75332	-0.33812
pH->abundance	0.539012	0.540953	0.149354	0.26931	0.78924
Fungal community	Original	Mean.Boot	Std.Error	perc.025	perc.975
N->P	0.822317	0.827789	0.035522	0.762089	0.892002
N->SOC	0.879913	0.883996	0.024716	0.82946	0.924798
N->community	0.578084	0.590788	0.208973	0.186744	0.97931
P->community	0.485536	0.450052	0.207626	0.136292	0.801826
SOC->pH	-0.58183	-0.56652	0.106434	-0.74623	-0.32458
pH->community	0.33272	0.332576	0.115113	0.122932	0.544701

A significant coefficient was identified that 0 was not covered between perc.025 and perc.975. N: nitrogen content (TN and ammonium), P: phosphorus content (TP and AP), SOC: organic carbon content, abundance: fungal abundance, community: fungal community. The fungal community was represented by the first two axes of PCoA.

Table S8 Results of fungal network robustness measured by the Paired t-test

Comparison pair	t	df_{total}	Two-tailed P-value
BS - AC	0.5970	200	0.5589
BS - LC	2.8277	200	0.0121*
BS - MC	2.5354	200	0.0220*
AC - LC	2.0376	200	0.0585
AC - MC	2.9207	200	0.0100**
LC - MC	5.2531	200	0.0001***

Robustness was calculated by random removing 50% nodes of network with 100 permutations. BS: bare sand, AC: algal crust, LC: lichen crust, MC: moss crust. ***represents $P < 0.001$, **represents $P < 0.01$,*represents $P < 0.05$.
